# Supplementary material for: Phenotypic characterization of cryptic species in the fungal pathogen Histoplasma
Source: bioRxiv. 2024 Jan 8:2024.01.08.574719. Preprint. [Version 1] doi: 10.1101/2024.01.08.574719 (PMC10802462; doi:10.1101/2024.01.08.574719)
Supplement: 1 [file NIHPP2024.01.08.574719v1-supplement-1.pdf]

bioRxiv preprint doi: https://doi.org/10.1101/2024.01.08.574719; this version posted January 11, 2024. The copyright holder for this preprint (which was not certified by peer review) is the author/funder, who has granted bioRxiv a license to display the preprint in perpetuity. It is made available under aCC-BY 4.0 International license.

SUPPLEMENTARY MATERIAL

TABLE S1. AIC values for the best fitting dose-response and linear functions for the growth curve of each *Histoplasma* species.

	AIC	
**Species**	**Linear**	**Dose-response**
H. suramericanum	-5.038041	-20.2201
H. ohiense	16.69309	-8.40372
H. mississippiense	9.61138	-8.56707
H. capsulatum ss	13.66652	-41.2338
Africa	1.258701	-71.5962

TABLE S2. Effect of different optimization values for the dose-response functions.

bioRxiv preprint doi: https://doi.org/10.1101/2024.01.08.574719; this version posted January 11, 2024. The copyright holder for this preprint (which was not certified by peer review) is the author/funder, who has granted bioRxiv a license to display the preprint in perpetuity. It is made available under aCC-BY 4.0 International license.

**Species**	**Starting Parameters**	**AIC**	**Inferred Parameters**
H. suramericanum	*a*=0.97, *b*=10, *c*=100, *d*=1.8	-20.22012	*a* 0.05461
			b 10.00000
			c 99.70641
			d 1.54093
H. suramericanum	*a*=0.97, *b*=10, *c*=10, *d*=1.8	38.83496	*a* 0.4198
			b 10.0000
			c 160.0000
			d 2.0000
H. suramericanum	*a*=0.97, *b*=12, *c*=20, *d*=2	-20.22012	*a* 0.05460
			b 10.00000
			c 99.70616
			d 1.54093
H. ohiense	*a*=0.97, *b*=10, *c*=30, *d*=2	-8.403715	*a* -0.01128
			b 5.13518
			c 76.21559
			d 2.26046
H. ohiense	*a*=0, *b*=50, *c*=80, *d*=1	-8.403715	*a* -0.01128
			b 5.13521
			c 76.21554
			d 2.26045
H. ohiense	*a*=0.5, *b*=50, *c*=180, *d*=1	-8.403715	*a* -0.01128

bioRxiv preprint doi: https://doi.org/10.1101/2024.01.08.574719; this version posted January 11, 2024. The copyright holder for this preprint (which was not certified by peer review) is the author/funder, who has granted bioRxiv a license to display the preprint in perpetuity. It is made available under aCC-BY 4.0 International license.

				b 5.13516
				c 76.21556
				d 2.26046
H. mississippiense	*a*=.97, *b*=10, *c*=30, *d*=6		-8.567065	*a* 0.00401
				b 5.58656
				c 110.24761
				d 2.08720
H. mississippiense	*a*=.07, *b*=10, *c*=130, *d*=6		-8.567065	*a* 0.00401
				b 5.58656
				c 110.24761
				d 2.08720
H. mississippiense	*a*=1, *b*=50, *c*=130, *d*=2		-8.567065	*a* 0.00401
				b 5.58656
				c 110.24761
				d 2.08720
H. capsulatum ss	*a*=0, *b*=1, *c*=50, *d*=2		-41.2338	*a* 9.231e-05
				b 5.150
				c 7.449e+01
				d 2.138e+00
H. capsulatum ss	*a*=0, *b*=10, *c*=100, *d*=2		-30.06304	*a* 0.11000
				b 5.76204
				c 76.18363
				d 2.11889

bioRxiv preprint doi: https://doi.org/10.1101/2024.01.08.574719; this version posted January 11, 2024. The copyright holder for this preprint (which was not certified by peer review) is the author/funder, who has granted bioRxiv a license to display the preprint in perpetuity. It is made available under aCC-BY 4.0 International license.

H. capsulatum ss	*a*=0, *b*=10, *c*=150, *d*=2	-13.03278	*a* -0.06824
			b 3.02782
			c 81.79755
			d 2.50000
Africa	*a*=6, *b*=4, *c*=30, *d*=3	-33.68027	*a* 6.738e-03
			b 3.384e+00
			c 1.000e+02
			d 2.207e+00
Africa	*a*=1, *b*=4, *c*=30, *d*=3	-71.59624	*a* 0.002906
			b 5.196654
			c 86.218632
			d 1.870503
Africa	*a*=1, *b*=40, *c*=30, *d*=3	-4.478411	*a* -0.1266
			b 1.5113
			c 200.0000
			d 4.4355
